# Nomina anatomica-unde venient et quo vaditis?

**DOI:** 10.1007/s12565-024-00762-w

**Published:** 2024-04-05

**Authors:** Michael L. Pretterklieber

**Affiliations:** Division of Macroscopic and Clinical Anatomy, Gottfried Schatz Research Center, Medical University of Graz, Auenbruggerplatz 25, 8036 Graz, Austria

**Keywords:** Anatomical terminology, Anatomical nomenclature, Medical history, Greek anatomical terms, Latin anatomical terms

## Abstract

As the title indicates, this article deals with the origins of anatomic terminology and its development up to the present day. The first attempt to name anatomical structures in animals and humans date back to Alkmaion, i.e. to the fifth century BC. Further work has been done at the same time by the Hippocratics and about 100 years later by Aristotle. As the Alexandrians Erasistratos and Herophilos first in history dissected human bodies, they expanded the anatomical terms. Until Celsus (around Christ’s birth) and even later on, anatomical terminology was almost exclusively based on the Greek language. Thus, Celsus and not—as frequently done—Galenos has to be called the father of Latin-based anatomical terminology. Due to several translations including Arabic, first periods of proverbial Bable resulted. Return to systematic order was achieved finally by Andreas Vesal (1514/15–1564) and Caspar Bauhin (1560–1624). But again due to translations into several national languages, the uniformity of the anatomical nomenclature was undermined. Thus, by the end of the nineteenth century, in 1895 the newly founded Anatomische Gesellschaft created a uniform terminology, the Basle Nomina Anatomica (BNA). Although it has been revised several times, it is still the very basic of human anatomical terminology. Recently, an attempt was made to replace it by English translations of the original Latin (and also still Greek) terms to mainly get machine-readable denominations. As this will result again in non-uniformity of terminology, the Anatomische Gesellschaft proposes a version of the latest, generally accepted terminology, based on the Latin terms but incorporating recent developments.

## Historical development of the anatomical nomenclature

### Anatomical description apparently starts with Alkmaion

**Fig. 1:**
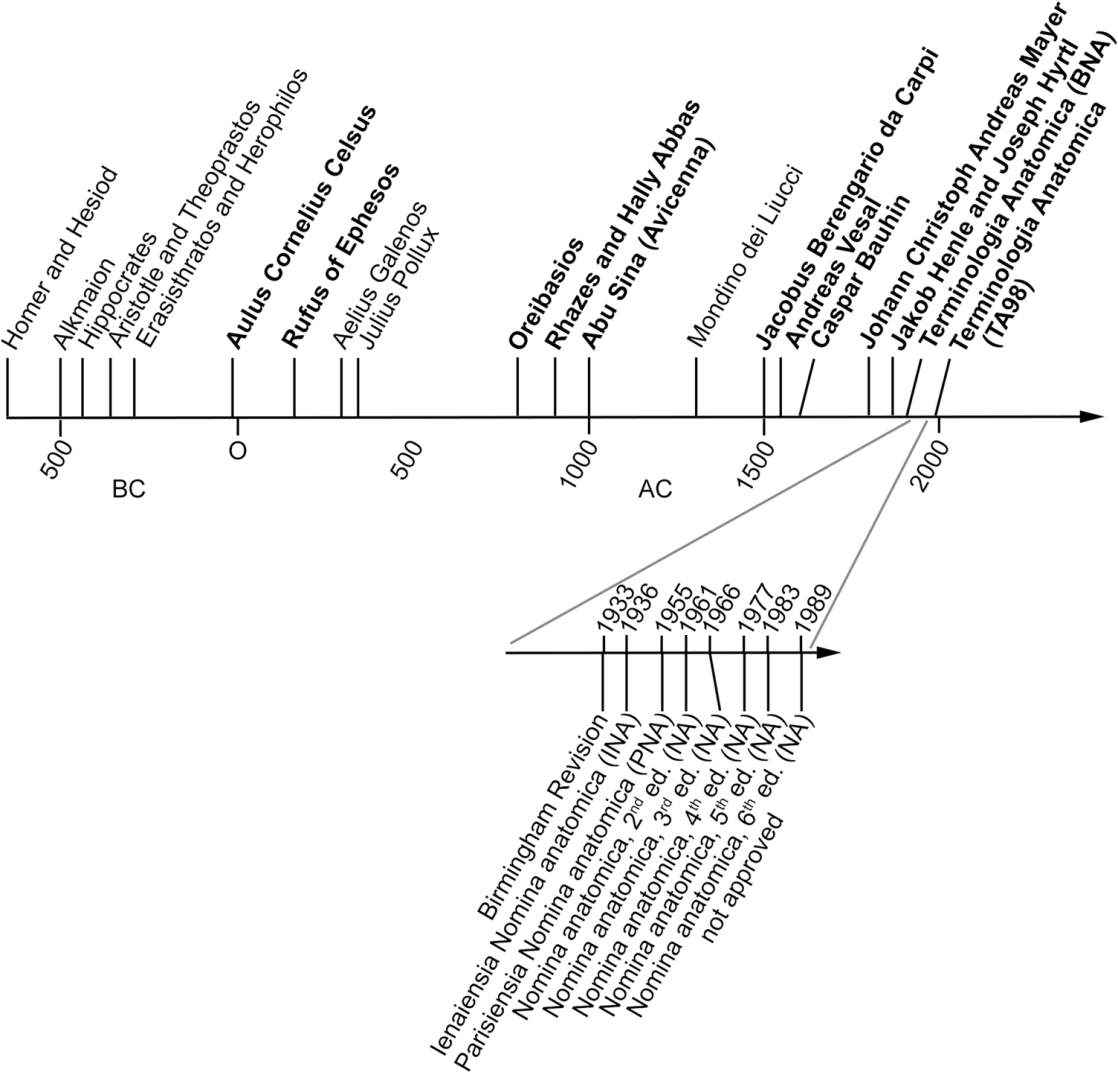
Timeline of the development of anatomical nomenclature. This timeline starts with Homer and Hesiod living around 700 BC, as from their works several anatomical terms with mythic origin were generated. In the further course of time, authors important for trading ancient texts and/or establishing a Latin-based terminology are indicated in bold letters. The extension in the lower half shows the time series of the various revisions of the Basle Nomina anatomica. But despite of them, they are still the very base of anatomical terminology, thus highlighting the pioneering work of Wilhelm His and his co-workers

In Europe and thus today’s western world, naming of anatomical structure starts with the Greek philosopher (and perhaps also physician) Alkmaion (500–450 BC) (Huffman 2021). The corresponding timeline is given in Fig. 1. Alkmaion is said to be the founder of neuroscience as well as to use first the terms ʼAερτερια (Aerteria) and Φλεψ (Phleps) and thus to distinguish arteries and veins. Furthermore, also the discovery of the optic nerve is ascribed to him (Tubbs et al. 2019).

More detailed descriptions were given by Aristotle (Aristoteles 1831) in the fourth century BC and not—as someone may expect—by Hippocrates 1 century earlier. As already Aristotle stated in his text “Των περι το ζως ʽιστοριων” (Ton peri to zos historion, Historia animalium), the earlier physicians did not have any exact anatomical knowledge. Aristotle was the first to dissect animals, e.g. apes (Staden 1989). Moreover, texts ascribed to Hippocrates which may indicate anatomical knowledge (e.g., as Περι φυσιος ανϑροπων—Peri physios anthropon, De natura hominis, or Πρι ʼοσσεϖν φυσεως—Peri osseon physeos, De natura ossium or Περι ʼαρϑρων—Peri arthron, De articulis) have been identified as pseudo-hippocratic writings already in the early nineteenth century (Grimm 1785; Link 1815; Pettenkofer 1837). But regardless of whether Hippocrates or his son-in-law Polybos wrote Περι φυσιος ανϑροπων (Peri physis anthropon) or not, the wrong vascular anatomy described there indicates the absence of anatomical knowledge (Link 1815; Pettenkofer 1837; Pollak 1993). For it is told there that four pairs of vessels arise from the head and—after descending through the whole body—end up at the ankles and the midfoot regions. Based on this theory, several locations for phlebotomy are listed (Pettenkofer 1837). Principally, the manuscript of Polybos first introduces the Krasen-doctrine (Pollak 1993). Thus, the terms ‘Αιμα (Haima) for blood and Χολη (Chole) for bile first appeared there. According to Neuburger (Neuburger 1906), also the following terms date back to the school of Hippocrates: Διαφυσισ (Diaphysis), ’Επιφυσισ (Epiphysis), Περιοστεον (Periosteon), Διπλοη (Diploe). With Μυς (Mys) or Σαρξ (Sarx), the muscles are generally defined and among others, the terms Δελτοειδεος (Deltoeideos) or Πσοα (Psoa) have apparently Greek origin. As Hyrtl (Hyrtl 1880) stated, erroneously Riolan (Riolan 1649) has chosen the genitive form Πσοας ((Psoas) still in use. Nerves and tendons have not been distinguished at all and are commonly named Νευρον (Neuron) or Τονοι (Tonoi) (Neuburger and Pagel 1902).

Other anatomical terms have been invented by Aristotle in another manuscript named “ Περι ζωϖν μοριϖν” (Peri zoon morion, De partibus animalium) (Aristoteles 1831). First of all, he used the word “Οργανον (organ)” to describe parts of the body with defined scope. Therefore, this term is kindred to another Greek word, namely “Εργον (ergon)” which means work. Moreover, he realized that animal bodies are composed of different fabrics called θηρα, (Thera). Several other general terms such as ‘Αιμα (Haima) for blood, Μυελος (Myelos) for marrow, Γονη (Gone) for knee, Χολη (Chole) for bile, Σαρξ (Sarx) for flesh and Φλεψ (Phleps) for vein or vessel in general are also to be found in this text. As already stated above, ‘Αιμα, Χολη and Φλεψ were apparently known before. But Aristotle, in addition, also first described the higher senses of smelling, seeing, tasting, hearing and the equilibrium apparatus. Due to the Greek origin of denominating parts of the body, several Greek words are still in use. Hence, despite of the rule given by His already in 1895 (His 1895) that anatomical terms should be of Latin origin and linguistically correct, terms like ʼΑτλας (Atlas), ʼΑξις (Axis), Γαστηρ (Gaster), Λαρυγξ (Larynx), Μεσος, original Μεσαραιον (Mesos, Mesaraion) (Hyrtl 1880), Μηνιγξ (Meninx), ϑραχυς ʼAερτερια (trachys Aerteria, Trachea, rough airpipe), ʼΑερτερια (Aerteria, Arteria, airpipe containing pneuma) are well-known and persistent.

Theophrastos, former member of Plato’s academy and later the most important pupil of Aristotle whom he followed as scholarch of the peripatos, issued a series of physiological writings (Gruner 1782). Together with Aristotle, he has introduced the term ʼΙρις (Iris) into botany thus describing the various, rainbow-like colours of flags. However, in human anatomy the term was entered later on by Rufus of Ephesos (around 100 AC, see below) (Karenberg 2005).

In addition, anatomical terms are partly of even mythic origin (Karenberg 2005). In alphabetical order, 12 examples are given in Table 1. As noted there, they are partly as old as the epics of Homer (Homer 2008) or the Θεογονια (Theogonia), of Hesiodos (Hesiodos 1908) describing the genesis of the Olympic gods. Both are currently thought to have lived in the seventh century BC. But interestingly, most of them have been taken from the poems of Publius Ovidius Naso (43 BC–17 AC) written in the classic Roman period (Ovidius Naso, 1914, 1971) or even introduced as late as in the sixteenth and eighteenth century (Bauhin 1588; Croissant de Garengeot 1742).

**Table 1:** Twelve examples of anatomical terms with mythic origin (from Karenberg 2005)

Term	Original meaning	Origin
Achillis tendon (Chorda Achillis)	Greek heroe	Homer, Ilias (Homer 2008)
Am(m)ons horn (Cornu Ammonis)	Theban god Amun [also written Am(m)on]	René Croissant de Garengeot (Croissant de Garengeot 1742)
Arachnoidea	Arachne, Greek spinner	Ovid, Metamorphoses (Ovidius Naso 1914)
Atlas	One of the titans	Hesiodos, Theogonia (Hesiodos 1908)
Hippocampus	Draught-animal of Poseidon’s conch / sea-horse	Hesiodos, Theogonia (Hesiodos 1908); (Aranzi 1587; Bir et al. 2015)
Hymen	Greek god of wedding and marriage; wedding song	Hesiodos, Theogonia (Hesiodos 1908)
Iris	Messenger of Greek god Hera; personified rainbow	Ovid, Metamorphoses (Ovidius Naso 1914); Rufus of Ephesos (Daremberg and Ruelle 1879)
Lymphe	Clear and clean spring water	Ovid, Metamorphoses (Ovidius Naso 1914)
Morphe	Morpheus, one of the sons of Greek god Hypnos	Ovid, Metamorphoses (Ovidius Naso 1914)
Philtrum	Philtron = philtre, aphrodisiac	Rufus of Ephesos (Daremberg and Ruelle 1879); Julius Pollux (Pollux 1824)
Pomum Adami	Hebraic “tappuach ha adam”—bump or apple on a man	Caspar Bauhin (Bauhin 1588)
Terminus	Youngest son of Greek god Saturn; identification mark	Ovid, Fasti (Ovidius Naso 1971)

Simultaneous to the life of Aristotle, first attempts to perform real human anatomy occurred. They took place in Alexandria founded in 331 BC by Alexandre the Great. In the Μουσαιον (Mousaion) of Alexandria, the Greek physicians Herophilus of Chalkedon (~ 335–~ 280 BC) and Erasistratos of Keos (31–250 BC) became the first human anatomists. Unfortunately enough, they performed their studies not only on dead but also on living human beings, i.e. they demonstrably vivisected prisoners lying under a sentence of death. Their cruel and inhuman work was allowed by the monarchs Ptolemaius I Soter followed by his son Ptolemaius II Philadelphos and already condemned in Roman times (Lee 1831; Tertullianus 1844). However, from the fragmentary preserved writing ʼΑνατομη (Anatomé) by Herophilos’, the following terms have been traded until today:

- Μητρα (Metra) used in Latinised composed terms to describe both the different layers of the uterine wall (peri-, myo- and endometrium) and the uterine mesentery (mesometrium);
- Νευρον (Neuron) invarably for nerve and not—as before but even later (see below)—for tendons; and
- ʼΕνκεφαλον (Enkephalon, Latinised Encephalon) for the brain.

### Successors and a translator in the classical and medieval period (~ 25 BC–1500 AC)

Before going into details from this period, it has to be said that “Terminus” is again of mythic origin (Karenberg 2005). Terminus, as likewise told by Ovidius in his poem “Festa”, representing a calendar of Roman festive days, was the youngest son of god Saturn. Among his brothers and sisters, he was the only one his mother managed to be preserved from being slung by his father. Therefore, he was able to beguile his father into vomiting and save the lives of his relatives. Thus, he finished this cruelty and was worshiped together with Jupiter considered as protector of boundaries. In medieval times, the original meaning of terminus changed into “confined, exact definition”. In short, Terminus is originally characterized as follows (English translation given by the author):

Terminus, ut veteres memorant, immotus in aede

restitit et magni cum Iove templa tenet.

Nunc quoque, se supra ne quid nisi sidera cernat,

exiguum templi tecta foramen habent. (Ovidius Naso 1971).

Terminus, as the elderly tell, remains immobile in the house

and together with Jupiter, the Great owns a temple.

To be sure he will only see the stars above him

the roof of the temple a small opening has.

Despite of Roman hegemony, Greek medical writings were only translated into Latin by Aulus Cornelius Celsus (~ 25 BC–~ 50 AC) (Lee 1831), thus this language was still only partly used in Anatomical terminology. Others like Rufus of Ephesus (~ 80–~ 150 AC) (Daremberg and Ruelle 1879), Julius Pollux (lived around 190 AC) (Pollux 1824), and Oreibasios (~ 325–403 AC) (Oreibasios 1556) retained Greek terminology. Thus, Aelius Galenos (130–200/201 AC) (Galenos 1525b; Töppli 1904) who is also said to represent a root of contemporary anatomical terminology (O’Rahilly 1989; Sakai 2007) still published in Greek. His writings have first been translated into Latin and issued as late as in 1529 (Galenos 1529; Töppli 1904). Nevertheless, he occupies an outstanding position in medical history by compiling and thus preserving the knowledge of his ancestors (Töppli 1904).

Aulus Cornelius Celsus is known as the first—and as one has to admit—only author of this period to write medical texts in Latin. In chapter one of the fourth book of his “De medicina libri octo” entitled “De interioribus sedibus corporis humani” he gave an overview of internal structures of the body (Celsus 1465). Thereby, he introduced either Latinized versions of the original Greek terms or replaced them by appropriate Latin terms. He not only took over some of the original terms, e.g. Trachea, but also created descriptive translations (“aspera arteria”). He also introduced new terms, as e.g. Jejunum or Vertebra (Faller 1978). Examples of terms mostly still in use are alphabetically listed in Table 2. Thus, the work of this “Polyhistor Italicus” is to be called a base for nowadays anatomical terminology (Lee 1831).

**Table 2:** Examples of anatomical terms created by Celsus (1465)

Latin term	Greek original
Arteria	ʼΑερτερια (Aerteria) -airpipe
Aspera arteria	τραχυς ʼΑερτερια (trachys Aerteria, rough airpipe)
Auris	ʼΟυς, ʼΟτος (Ous, Otos)
Caecum	τυφλον ʼΕντερον (typhlon Enteron)
Caput	Κεφαλη (Kephale)
Carotidas	Καροτιδας (Karotidas)
Cartilago	Χονδρος (Chondros)
Cerebrum	ʼΕνκεφαλον (Enkephalon)
Cervix	Tραχηλος (Trachelos)
Cor	Καρδια (Kardia)
Coxa	ʼIσχιον (Ischion)
Crassium transversum	Κωλον πλαγιον (Kolon plagion)
Glandula	ʼΑδην (Aden)
Ilium	ʼειλεειν (eileein)
Intestinum	ʼΕντερον (Enteron)
Intestinum tenue	Εντερον λεπτον (Enteron lepton)
Jejunum	First used by Celsus (Faller 1978), later traded by Rufus as Νηστις (Nestis), see also Table 4 (Töppli 1904)
Lien	Σπλην (Splen)
Lingua	Γλοττις (Glottis)
Mamma	Μαστος (Mastos)
Medulla	Μυελον (Myelon)
Musculus	Μυς (Mys)
Nervus	Νευρον (Neuron)
Oculus	ʼΟφϑαλμος (Ophthalmos)
Omentum	ʼΕπιπλοον (Epiploon)
Os	ʼΟστεον (Osteon)
Ouretera	ʼΟυρητηρ (Oureter)
Palatum	ʼΟυρανικος (Ouranikos)
Peritoneum	Περιτοναιος (Peritonaios)
Porta	Πυλορον (Pyloron)
Pulmo	Πνευμων (Pneumon)
Rectum intestinum	Προκτον (Prokton)
Ren	Νεφρος (Nephros)
Septum	Διαφραγμα (Diaphragma)
Spina	Ραχις (Rachis)
Stomachus	Στομαχος (Stomachos)
Trachea	Τραχεα (Trachea)
Urina	ʼΟυρον (Ouron)
Uterus	Μητρα (Metra)
Vena	Φλεψ (Phleps)
Ventriculus	Στομαχος (Stomachos)
Vertebra	Σπονδυλος (Spondylos)
Vesica	Φυσαλις (Physalis) or Φυσημα (Physema)
Viscerum	Σπλαγχνος (Splanchnos)
Vulva	Δελφυς (Delphys)

Quite in contrast, Rufus of Ephesos (~ 80 to ~ 150 AC), although succeeding Celsus in lifetime, wrote in Greek and thus returned to the ancient Greek terms (Daremberg and Ruelle 1879). However, from his Περι ‘ονομασιας των του’ ανϑροπου μοριον (Peri onomasias ton tou anthropou morion, Concerning the names of the parts of a human, briefly ‘Ονομαστικον, Onomasticon, Compendium of names’) quite a series of Greek terms have survived. Examples of again still used terms are alphabetically listed in Table 3 (Töppli 1904).

**Table 3:** Examples of traded Greek terms by Rufus and their brief explanations (Töppli 1904)

Term	Explanation by Rufus
ʼΑκρομιον (Akromion)	A small ossicle as described by Eudemos (nowadays Os acromiale)
ʼΑμνιος (Amnios)	Thin and soft skin of a sheep as described by Empedokles
ʼΑορτη (Aorte)	Stem of all arteries, term created by Aristoteles
Βρογχιαι (Bronchiai)	Extensions of Bronchos into the lungs
Βρογχος (Bronchos)	Used synonymously to Trachys aerteria
Χωριον (Chorion)	Rough, outer covering of the child in utero rich of veins, gives rise to umbilicus with two arteries and two veins
Διαφραγμα (Diaphragma)	Membrane separating thorax and abdomen, synonym to Phrenes (see below)
ʼΕπιγλοττις (Epiglottis)	Near tongue, is stated above the Bronchos
ʼΕπιπλοον (Epiploon)	Arises from the curve of the stomach
Γαστηρ (Gaster)	Lies below the Diaphragma, also called “upper cave”
Γλουτοι (Glutoi)	Buttocks
ʽΥποϑεναρ (Hypothenar)	Region below the four fingers
ʼΙσχιον (Ischion)	Tendon arising from the socket of the hip joint or the hip joint as a whole
Καρδια (Kardia)	Hole below the thorax
Καρωτιδες (Karotides)	If pressed, Καρωδησ-Karodes, a dead faint is initiated
Καρπος (Karpos)	Root of the hand
Κλειτορις (Kleitoris)	Fleshy part in the midst of the pubic cleft; from κλειτοριζειν (kleitorizein) = indecent touching that part
Κνημη (Kneme)	Calf
Κοξξυξ (Kokzyx)	Lowermost part of the vertebral column formed like the beak of a cuckoo
Κωλον (Kolon)	“lower cave”, continuation of Nestis (see below)
Λαρυγξ (Larynx)	Head of Bronchos
Μεσαραιον (Mesaraion)	Link of intestines, contains lymph nodes; ʼΑραια (Araia) means tapering (of food)
Μεσεντεριον (Mesenterion)	Link of intestines
Μετακαρπος (Metakarpos)	Fixed part of hand distal to Karpos
Νηστις (Nestis)	Small intestine as a whole, nestis means fasting and arid
Νευρα (Neura)	Sensory nerves and strings arising from brain and spinal cord, otherwise also fibrous bands surrounding and connecting joints
ʼΟισοφαγος (Oisophagos)	The structure through which food and drinks slide downwards into the abdomen; synonymously used with Stomachos (see below)
ʼΟλεκρανον (Olekranon)	The peak supporting our reclining
ʼΟρεχεις (Orecheis)	The testes; original meaning = lump
ʼΟυρητηρες (Oureteres)	Paired, connecting the kidney and the urinary bladder
ʼΟυρηϑρα (Ourethra)	Tube for releasing urine and sperma
Παγκρεας (Pankreas)	Fatty and gland-like meat close to the origin of the intestine
Περικρανιος (Perikranios)	Covering of the skull bones beyond the skin
Περιοστεος (Periosteos)	Covering of other bones
Φαρυγξ (Pharynx)	All the wide room for engulfing
Φρηνες (Phrenes)	Synonym to Diaphragma (see above)
Στερνον (Sternon)	That part of the breast to which the ribs attach
Στομαχος (Stomachos)	Synonym to Oisophagos (see above)
Θηναρ (Thenar)	Space between the little finger and the thumb; according to Hippocrates the whole palm of the hand
Θυμος (Thymos)	Belongs to lymphactic nodes, situated at the head of the heart, close to the 7th cervical vertebra and the end of the trachea in front of the lungs
Τραχυς Αερτερια (Trachys Aerteria)	Airpipe, synonymously, used with Bronchos (see above)

Despite the fact that he is better known under his Latin name, Julius Pollux (recte Ιουλιος Πολυδευκησ, Ioulios Polydeukes) who lived around 190 AC, also wrote in Greek language. In contrast to the similar title, his ʽΟνομαστικον (Onomastikon) was a general lexicon covering not only medical topics. Thus, although he has been called a renowned authority in the field of naming the individual parts of the human body (Karenberg 2005), the second volume of his work dealing with anatomical terms only seems to be simply a compilation (Pollux 1824).

The last of the successors to be named here was Oreibasios (~ 325–403 AC). Born in Pergamon, he studied in Alexandria and became the most famous physician of his age. Thus, he was elected personal physician of the Roman emperor Julianus and also responsible for the imperial library. Furthermore, the emperor made him to compile the work of the earlier Greek physicians including Galenos and Rufus of Ephesos (Pollak 1993). His original 72 volume ʼΙατρικων συναγογων (Iatrikon synagogon, physicians’ meeting) has only been preserved in fragments (Oreibasios 1556; Bussemaker and Daremberg 1851, 1854, 1858, 1862). However, an extraction written for his son Eustathios called Συνοπσις (Synopsis) has also survived (Bussemaker and Daremberg 1873, 1876).

During the migration period, the Arabic empire extended into Spain. The Arabians highly appreciated any scientific writings and for better understanding translated them. Therefore, medical texts originally written in Greek and Latin have been preserved mainly in Arabic translations, e.g. by Rhazes, Hally Abbas, and Abu Sina (Wüstenfeld 1840). Moreover, among Arabic terms introduced into the description of the human body, a few still remain parts of anatomic terminology (Hyrtl 1879). Interestingly, they all denominate subcutaneous veins of the arm and leg, i.e.:

- the basilic vein which was originally called “al-basilik” (the internal) thus describing their course on the medial aspect of the arm;
- the cephalic vein, which derives its name from the Arabic “al-kifal” (colloquial “al-kefal”), meaning the outer. The current name only came about in modern times (1564) due to an incorrect translation into Greek (Hyrtl 1879). As this vein was often used for phlebotomy and this in turn was often used for treating headache, the interpretation as κεφαλικος (kephalikos) = belonging to the head was obvious;
- the saphenous veins on the leg, which derive their name from “al-saphein”—the hidden one.

Due to the reservations caused by the inhumane actions of Erasisthratos and Herophilos, it took until the turn of the thirteenth and fourteenth centuries before human anatomy was systematically practised again. The first physician who is known to practise human anatomy again was Mondino de’Liucci (´Luzzi), who lived between around 1275 and 1326 (Bynum and Porter 1993). He was able to end the 15 centuries of stagnation by restarting systematic dissection of dead human bodies. He wrote a small booklet called Anathomia, which can best be regarded as dissection guide. But, although he would have been able to study human anatomy hands on, he retained the old views of Galenos. This can be explained by the fact that Mondino, as a professor at that time, did not dissect himself, but left this to a butcher’s assistant. At the same time, he lectured the extant writings of Galenos and attempted to demonstrate what was described in the corpse as well as possible (Neuburger and Pagel 1902; Wölkart 1961). The apparently rather superficial treatment of human anatomy is reflected in the very limited size of the work. The oldest available print comprises only 38 pages (de Liucci 1482), and following prints range between 25 (de Liucci 1507) and 79 pages (de Liucci 1493). However, since Celsus he has been the first to write in Latin. But as he mostly used own and new terms, he already started the proverbial Bable in anatomical terminology (Neuburger and Pagel 1902). For example, apparently based on incorrect back translations from Arab language into Latin, he used spatula instead of scapula or furcula instead of clavicula. Nevertheless, as his booklet was an easy to read instruction manual, it has seen 25 editions and was used until the sixteenth century (Neuburger and Pagel 1902).

Moreover, based on this rather superficial knowledge but on—as he states—more than 100 dissected human corpses, Jacobus Berengario da Carpi (~ 1470–1530) took the next major step towards a standardised Latin nomenclature (Neuburger and Pagel 1903). In his “Isagogae breves, perlucidae ac uberrimae, in anatomiam humani corporis a communi medicorum academia usitatam”, he gave the first time an illustrated description of the inner organs (Berengario da Carpi 1523). Quite in contrast to Mondinos’ work, this book covers about 200 pages. The used terms are already the usual ones even now (e.g. Colon, Duodenum, Ileum, Jejunum, Ventriculus). He therefore moves on from the medieval period to the true reform of anatomy (Neuburger and Pagel 1903).

### First attempt of standardisation by Vesal

In the sixteenth century, two outstanding physicians are seen as true fathers of nowadays anatomical terminology, thus enabling their pupil to publish the first scientific book on human anatomy. They were Johannes Winther (Guenther) von Andernach (1505–1574) and Jacques Dubois (Jacobus Sylvius, 1478–1555) (O'Rahilly 1989; Kachlik et al. 2008). Based on their knowledge, in 1543 Andreas Vesal (1514/15–1564) issued his epoch-making “De corporis humani fabrica libri septem” (Vesal 1543). In this textbook thoroughly illustrated by wood engravings created by Jan Stephan van Calcar, Vesal replaced all former Greek and Arab terms by Latin ones already in use. But in contrast to his teacher Dubois, he used ordinal numbers instead of descriptive names to define the individual bones, muscles, vessels and nerves (Kachlik, et al. 2008). But as the same numbers were given several times, e.g. a muscle number one was defined in the upper arm and also in the hand, this apparently led to some confusion again. As Vesal’s outstanding monograph was published in Basel, Alban Thorer*,* the rector of the Basel University at that time, has compiled a short synopsis in German (Neuburger and Pagel 1903). In the introduction, he gave a first German description of the Greek and Latin terms used by Vesal. Moreover, one of the plates presented a very early form of interactivity using an overlay, i.e. the reader was enabled to superimpose an engraving of the vessels and inner organs over a display of the skeleton (Thorer 1543).

Forty-six years later, Caspar Bauhin (1560–1624), another pioneer of human anatomy who became Professor of anatomy at Basel in 1589, in his “De corporis humanis partibus externus tractatus” (Bauhin 1588) as well as in his “De corporis humani fabrica libri IV” (Bauhin 1590), reverted to Latin again. Moreover, he defined names for the individual bones, muscles and vessels, but interestingly retained the numbering of the nerves. Thus, the nomenclature was again inconsistent. However, Bauhin managed that the terms were more readily accepted by using illustrations (Sakai 2007). In addition, as the newly established names of muscles indicate their origin and insertion, this improved recognisability and they became easier to learn (Sakai 2007).

### Anatomical nomenclature until the time of Joseph Hyrtl

During the seventeenth and eighteenth centuries, anatomical nomenclature even became more complicated, first by adding a lot of new terms and second by using the respective national language instead of Latin (Sakai 2007). Two examples may serve as illustrations. In 1699, the outstanding Belgian anatomist Philip Verheyen (1648–1710), professor of anatomy in Leuven, issued his textbook in Latin (Verheyen 1699). In a very modern way, he used clear, thematically organised lists of the terms recognised at that time, accompanied by corresponding illustrations. However, in 1708 he published a German version of his textbook (Verheyen 1708), thereby creating explanatory translations of the original Latin and Greek terms. At least some of them are still synonymously used in the German speaking countries. On the other hand, he also created some new terms, e.g. “Spannadern” (tensioning vessels) for nerves, thereby illustrating the pure mechanistic understanding of nervous function dating back again to Galenos (Galenos 1525b; 1525a) and being still alive at that time (Casserius 1600). Another example is the monograph of Josias Weitbrecht (1702–1747) (Neuburger and Pagel 1903) on the anatomy of the joints from 1742 (Weitbrecht 1742). As it was the first and comprehensive syndesmology of the human body, about 30 years after his death it was also translated into German in 1779 (Weitbrecht 1779). Again, in the German version, the original terms are not only explained but also partly replaced by German terms, hence changing a consistent to an inconsistent terminology again.

Thus, as later citizised by Joseph Hyrtl (Hyrtl 1880), in that time medicine and especially anatomy failed to standardize their language as it has been done e.g. for the natural sciences by Linné (Linné, 1753a, b) introducing a binary terminology for plants. Nevertheless, with his four-volume anatomical textbook (Mayer 1783a, b, 1784, 1786), Johann Christian Andreas Mayer (1747–1801), professor of anatomy and botany at the University of Berlin, made the first attempt to standardize anatomical terminology (Hyrtl 1884). As in the description of individual structures he thoroughly referred to other anatomists, he unfortunately introduced a lot of synonyms again and even eponyms, e.g. “Highmors Höhle” or “antrum Highmori” or Sinus maxillaris (Mayer 1783b). This again led to inconsistency and thus a separate scientific branch of anatomy, anatomical synonymy had been established (Hyrtl 1880). A special lexicon in which all known synonyms were listed was needed so that the anatomists with their different locations could communicate at all (Schreger 1805; Pierer 1816). As Joseph Hyrtl (1811–1894) criticised this inconsistency several times (Hyrtl 1870, 1879, 1880, 1884), he usually used only one term for an anatomical structure in his textbooks (Hyrtl 1862, 1870). In annotations, however, he referred to older and synonymously used terms (Hyrtl 1870). But Hyrtl did not only criticise inconsistency in anatomic nomenclature by the unnecessary use of synonyms. In addition, in his three monographs (Hyrtl 1879, 1880, 1884) he pointed out several mistakes caused by apparently inadequate language skills. Some examples of misnomers still alive are given in Table 4. In addition, Table 5 shows some examples of—as defined again by Hyrtl—“barbarian use of Latin”. In Table 6, characteristic examples of misspellings of Greek terms due to incorrect pronunciation are provided (Hyrtl 1879). Despite his intensive involvement with the existing problems of anatomical nomenclature, Hyrtl did not see himself as an individual in a position to reform it. His Onomatologa anatomica (Hyrtl 1880) was also—contrary to a recently held opinion (Kachlik et al. 2008)—not intended as the first approach to solving this problem. Instead, he determined that an ad hoc committee of linguistically competent anatomists was needed to solve this pressing problem, who would have to form an Academia della crusca anatomica with philological assistance. Hyrtl thereby alluded to the Accademia della Crusca founded in Florence in 1583. Still existing, it is today considered the oldest language society and has set itself the task of studying and preserving the Italian language. This is why it published the first dictionary of Italian as early as 1612. The name “Crusca” is derived from “crusconi”, i.e. bran flakes. The few founding members who wanted to separate the wheat from the chaff linguistically saw themselves as such (Anonymous 2023).

**Table 4:** Examples of misnomer from the past still alive (from Hyrtl 1880)

Term	Real meaning
canalis tubarius	Trumpet forming canal
cardiac nerve	Cardially ill (= cardiac) nerve
Cilia	Eyelid
endothel	Internal verruca
epigastrium	Abdominal wall
lamina cribrosa	Lamina rich of sieves
musculus vastus	Deserted muscle
orbita	Rail
os palatinum	Bone of palatium mountain, imperial bone
profundus	Bottomless deep
thalamus	Chamber (not hill!)
vasa lymphatica	Mentally ill vessels
vulva (recte volva!)	Uterus of pig

**Table 5:** Examples of barbarian Latin translations (from Hyrtl 1879)

Term	Desired meaning	Correct term
Acinus	Uvula	Uvula
Acceptabulum	Socket of hip joint	Acetabulum
Antecardium	Trigone of the pericard	Area interpleurica inferior
Anus	Rectum	Rectum
Arteria	Air pipe	Trachea
Brachiale	Carpus	Carpus
Bregma	Anterior fontanelle	Fonticulus anterior
Coax	Femur	Coxa or Femur
Cochlea	Ear conch	Auricula
Coelum	Hard palate	Palatum durum
Concha	Pudendal cleft	Rima pudendi
Corda or Chorda	Tendon	Tendo
Epiglottis	Voice box	Larynx
Extremitas	Limb	Membrum
Foliolum	Anterior fontanelle	Fonticulus anterior
Folium	Greater omentum	Omentum majus
Fons	Medial ocular angle (where the tears accumulate)	Angulus oculi medialis
Inguen	Male external genitalia	Organa genitalia masculina externa
Lacertus	Muscle	Musculus
Mediastinum	Mediastinal part of pleura parietalis	Pars mediastinalis of Pleura parietalis
Metapedium	Middle part of the foot	Metatarsus
Nervi	Ligaments of joints	Ligamenta
Nodus	Joint	Articulatio
Omenta	Meninges	Meninges
Os coxae	Femur	Femur
Os femoris	Os coxae	Os coxae
Os parietale	Temporal bone	Os temporale
Parotium	Lateral ocular angle	Angulus oculi lateralis
Porternarius	Pylorus	Pylorus
Restricta and recepta (instead of rascetta)	Carpus	Carpus
Scrotum (cordis)	Heart sac	Pericardium
Thorax	Sternum	Sternum
Vena	Artery	Arteria
Vulva	Uterus	Uterus

**Table 6:** Examples of apparently onomatopoetic spelled Greek terms (from Hyrtl 1879)

Term	Correct spelling
Anathomia	Anatomia
Cradia	Cardia
Dyaphragma	Diaphragma
Faringa or Farix	Pharynx
Gastrocurmia or Gastrognymius	Gastrocnemius
Glangula	Ganglia
Ithmides	Ethmoideus
Laringa or Larix	Larynx
Mescrenum	Mesenterium
Obtalmia	Ophthalmica
Obticus	Opticus
Olectranum or Olenoctranum	Olecranon
Orthi	Aorta
Panagra	Pancreas
Permeum	Perineum
Pileron	Pylorus
Praeputium	Proposthion (προ = pro = anterior and ποσϑη = posthe = penis)
Spondylus	Spondylos

## Attempts of standardisation anatomical terms during the last 2 centuries—the Nomina anatomica

In the introduction to the Basle Nomina anatomica (His 1895), Wilhelm His (1831–1905) emphasized the importance of Jakob Henle (1809–1885) in the standardization of anatomical nomenclature. For his three-volume textbook of human anatomy (Henle 1871a, b, 1872, 1873, 1876), Henle selected a specific term for each anatomical structure. However, since at the same time he has given in footnotes all the terms used at that time as synonyms, no standardization could be achieved in this way. Thus, the only progress was that he broke with the use of eponyms, for using them implies historical injustices. Moreover, as Henle offered synonyms it was again left to the individual teacher and researcher to choose a preferred term. As a result, some followed Henle’s selection while others did not, and the consequence was that each university had its own anatomical terminology. Students became confused about it and even doctors were only able to understand publications written using the anatomical nomenclature they had learned.

To overcome this obvious deficiency, the Anatomische Gesellschaft established a Nomenclature Commission at its 1889 meeting in Berlin, i.e. an Anatomia della crusca as suggested by Hyrtl again in the same year in the 20th edition of his textbook on systematic anatomy (Hyrtl 1889). The commission was headed by Albert von Koelliker and the members were Oscar Hertwig, Wilhelm His, Julius Kollmann, Friedrich Merkel, Gustav Schwalbe, Carl Toldt, Wilhelm von Waldeyer-Hartz, and Karl von Bardeleben (His 1895). During 6 years of intensive discussion, they established some fundamental rules, the most important of them are briefly summarized here (His 1895; Eycleshymer et al. 1917):

1. each structure shall be given only one unique and unmistakeable name;
2. the terms must be Latin and linguistically correct, short and simple;
3. related terms shall be similar (e.g., Femur, A. femoralis, N. femoralis);
4. the terms shall represent memory signs and not any explanations or speculative interpretations;
5. adjectives shall generally have their opposites assigned (e.g., dexter/sinister, major/ minor, superficial/ profound).

Despite all efforts, some longer terms have to be kept alive, e.g. M. sternocleidomastoideus. As another example, it was not possible to replace the Foramen spinosum by Foramen meningeum medium. Due to the ongoing use in clinical practice, eponyms were not completely eradicated, but reduced to only 20. According to the rules established by the zoological nomenclature commission (Blanchard 1893), 3 were used in osteology, 6 in myology and 11 in angiology and added in brackets to the respective terms (Krause 1893). Overall base of the hence created “Nomina Anatomica” (His 1895) was the anatomical textbook of Carl Gegenbaur (Gegenbaur 1883) because it offered the most up-to-date and thoroughly edited presentation of systematic anatomy (Krause 1893; His 1895). As it was adopted by the 1895 annual meeting of the Anatomische Gesellschaft at Basel, it was therefore called the Basle Nomina Anatomica, abbreviated BNA. In addition to the above mentioned advances, some incorrect terms have been replaced, e.g. thoracic vertebrae instead of dorsal vertebrae, for all the vertebrae are situated dorsally in the human body (O'Rahilly 1989). Furthermore, simply by eliminating synonyms, the number of terms has been reduced from about 50,000 to roughly 5,000 (Kachlik et al. 2008). However, all in all the BNA was a very conservative compromise with only a few new terms established. Moreover, the Anatomische Gesellschaft only recommend to use it and as it was seen as affair of this (mainly German speaking) society, it was international only partially accepted. This was despite of the given invitation for international cooperation and the authorisation to use their equivalents in the respective national language instead of the original Latin terms (His 1895; O'Rahilly 1989).

Although the BNA was soon adopted in America, it was rather ignored in Italy and Great Britain. By 1918, the Anatomical Society of Great Britain and Ireland decided that a revision of the BNA is necessary and should be based on the 10th edition of Quain’s Elements of Anatomy (Schäfer and Thane 1891). Work was started in 1928 and finished in 1933, resulting into the Birmingham Revision (BR) of the BNA (Anat.-Soc.-Great-Britain-and-Ireland 1933; O'Rahilly 1989). It was very close to the BNA, but had also some new and thereafter commonly accepted terms, e.g. facial artery instead of external maxillary artery (O'Rahilly 1989).

Almost parallel, the Anatomische Gesellschaft in 1923 started an own revision of the BNA. This was accepted at the 1936 annual meeting at Jena and thus named the Ienaiensia Nomina Anatomica (INA) (O'Rahilly 1989; Kachlik, et al. 2008). To get acquainted with the altered terms, a list comparing the BNA and INA versions was provided (Kopsch 1937). As said there, 5291 terms listed in the BNA were reduced to 5124. Thus, 670 old terms were exclused, but 498 terms newly introduced. Only slightly changed were 1105 and more significantly modified 146 terms. Some examples for alterations are ilicus instead of iliacus, or meningicus replacing meningeus. As in the BR, the A. maxillaris externa was now called A. facialis and instead of A. anonyma, the term Truncus brachiocephalicus was introduced. Moreover, seemingly all eponyms have been cancelled (Kopsch 1937).

After the interruption caused by World War II, the International Federation of Associations of Anatomists (IFAA), founded already in 1903, in their 1950 congress held at Oxford established the International Anatomical Nomenclature Committee (IANC) which should revise both the BR and INA (O'Rahilly 1989). This work started only in 1952. It should result in a Latin anatomical nomenclature and—as the INA was entirely disapproved—be based on the BNA again. This revision of the BNA was finally approved as the first official international anatomical terminology at the sixth congress of the IFAA in Paris 1955 and therefore named the Parisiensia Nomina Anatomica, abbreviated PNA (Sakai 2007; Kachlik et al. 2008). Again, a comparison of the BNA, INA and PNA soon became available (Knese 1957). The PNA was first revised as soon as in 1961 and from this 2nd edition onwards named Nomina Anatomica (NA). A revised 3rd edition followed in 1966 and its 4th edition was issued in 1977. In 1983, the 5th edition of the NA was published. Thereafter, apparently there was a disagreement between the IFAA and the IANC. Thus, the 6th edition issued by the IANC in 1989 was neither approved by the IFAA nor accepted by anatomists around the world. To solve that problem, the IFAA decided to nominate a new commission called Federative Committee on Anatomical Terminology (FCAT). In 1998, that Committee issued the new nomenclature now called Terminologia anatomica or International anatomical nomenclature, abbreviated TA98. As the title suggests, it has become a bilingual nomenclature offering the anatomical terms both in Latin and English (Sakai 2007; Kachlik et al. 2008). It is still mainly based on the BNA of 1895 and—as already offered at that time—has been translated into many other languages as, e.g. Japanese, Chinese, French and even Esperanto (Sakai 2007). In contrast to the BNA, all eponyms are now eliminated.

Since all of these nomenclatures are pure alphabetical lists, in 1967 the German anatomist Heinz Feneis first issued an illustrated version of the TA called “Anatomische Bildnomenklatur”. This pioneering work has followed all the revisions of the NA and finally—based on the TA98—has reached its 11th edition (Dauber 2019). It has been also translated into several languages and the English version—in the meanwhile also in its 5th edition—is entitled “Pocket Atlas of Human Anatomy”(Dauber 2007). In between, Wolfgang Dauber has taken over the work of the late Heinz Feneis.

In 2017, Paul E. Neumann and co-workers published an article criticizing the exceptions to the rules of the international codes of nomenclature in the natural sciences. They stated that to the existing seven rules of anatomical nomenclature (the main five of them are listed above) another five as numbered by the authors should be added which are as follows (Neumann et al. 2017):

8. that each name must be unique;
9. that each name shall consist only of nouns and adjectives;
10. that each name shall have only one noun in nominative case;
11. that the standard word order shall have nouns following the noun they modify, and adjectives immediately following the noun they modify;
12. that nouns in genitive case are generally preferable to adjectives when the modifier means “of” an entity, rather than “pertaining to” an entity.

Based on these rules (Fraher 2018), new anatomic names called Regular Anatomy (RA) terms were created which were originally thought to be presented along with the official terms of the TA98. But in 2019, the now called FIPAT (Federative International Programme for Anatomical Terminology), first published a 2nd edition of the TA (TA2) (FIPAT 2019) which was ratified by the IFAA in 2021. In this version, the RA terms were placed in front and the original terms were only found under synonyms or even under “other”. Therefore, starting with Dutch and Flemish Anatomists in 2020 (ten Donkelaar and Gobée 2020), most European anatomists heavily criticized the genesis and overall arrangement of the TA2. However, it was realised that the TA2 also offers some improvements.

To solve that problem and to initiate a new discussion process, the Anatomische Gesellschaft in 2022 established a tripartite working group headed by Christoph Viebahn and significantly supported by the preparation and co-operation of Luis Filgueira. The first subgroup dealt with general anatomy and the musculoskeletal system, the second with the inner organs and the third with neuroanatomy and sensory organs. Members of the groups (including the author) represented anatomical institutes from central and Eastern Europe. The main tasks were to find reasons against the use of TA2 as well as for using specific terms first given by the TA2. There was a strict schedule, for the results should be presented at the annual meeting held at Berlin in September 2022. Based on the final report of the working group presented there, the next steps were determined. Following extensive general editing by the chairman of the working group, Christoph Viebahn (also assisted by the author), the final version of a revised TA98 now called “Terminologia anatomica 2023 of the Anatomische Gesellschaft—International Anatomical Terminology (TA2023AG)” was adopted at the next annual meeting in Würzburg in September 2023. As the general assembling of the Anatomische Gesellschaft recommended to use this revision, especially for anatomical textbooks and other teaching material, it has been made available in pdf format to all members and will also be downloadable from the homepage of the Anatomische Gesellschaft in the near future.

## Strengths, weaknesses, opportunities, and threats (SWOT)—analysis of the second edition of the Terminologia anatomica (TA2)

Finally, as a member of the working group, I briefly summarize the overall impression and also my experience in a kind of SWOT analysis. The initial idea of harmonising the anatomical terminology more closely with the international codes of nomenclature of the natural sciences (Neumann et al. 2017) may have been successful. Nevertheless, it has neither improved readability nor simplified the terms. Moreover, some ideas clearly contradict the given rules. For replacing the Latin word “os” meaning bone or mouth in the latter case by the Greek word “stoma” is against the accepted principle that only Latin terms should be used (His 1895; Neumann, et al. 2017). Second, omitting the term “Musculus” for muscles that are named to indicate their function is neither useful nor beneficial. For it makes it impossible to create an organised subject index. This is because it results, for example, in the Extensor indicis ending up under the letter E and the Supinator under S, whereas the other muscles are listed under M. Moreover, on a closer view it turned out that the rule has not been followed thoroughly. For e.g. the term Musculus opponens pollicis clearly indicates that this muscle will oppose the thumb and thus should have be named “Opponens pollicis”. Another example is given with the both Musculi obturatorii externus et internus. Although the Latin word “obturare” does not describe any action of these muscles on the hip joint, they lack the term Musculus.

Moreover, the word order given by rules no. 10 and 11 result into rather erroneous terms. For example, Musculus extensor carpi radialis brevis clearly indicates that this muscle acts as extensor and radial deviator in the wrist joints and is the shorter of two. The new term “Externsor radialis brevis carpi” at least for a newcomer arises some questions: does it extend the radius, is it only to be found in short radii or in case of a short carpus? Thus, it does not improve the existing nomenclature (i.e. the TA98 or TA2023AG) and should be avoided. Therefore, the TA2023AG incorporates the myological terms of the TA98.

This misleading word order has even more far-reaching consequences for the names of the nerves supplying the muscles. For example, the Ramus musculi extensoris carpi radialis longi is changed to Ramus extensoris radialis brevis carpi. Questionable, if this should be a short branch of the hand stretching the radius or a branch of the hand slightly stretching the radius? Thus, against the statement of Neuman et al. (Neumann, et al. 2017), word order is important for understanding and should not be corrupted. These unnecessary failures remind me more to former errors criticised by Hyrtl (Hyrtl 1879, 1880) which are exemplified in Table 4 and 5 than to a reliable nomenclature which has to be both anatomically and linguistically correct. Moreover, the principal question arises, why not—instead of creating such misleading terms—the already available and fully accepted Terminologia Neuroanatomica (TNA) has been used (ten Donkelaar and Gobée 2020). As this is a fully reliable terminology, it has been incorporated into the TA2023AG.

The reintroduction of Latin and even English synonyms represents a major step backwards to the time before the BNA. As this was one of the main goals in creating the BNA (His 1895) which is apparently still the very basis of anatomical nomenclature, it should have been avoided anyway. Furthermore, the introduction of synonyms apparently contradicts the principle of unique naming, which was demanded by the proponents themselves (Neumann, et al. 2017). And by erasing two of the six columns introduced for synonyms, the readability of the terminological lists will be very much improved. Moreover, combining the columns for UK- and US-English terms which are only partially different, will improve the clarity even more. It would be quite sufficient to separate individual different terms with a slash.

Furthermore, in dealing especially with chapters 1 and 2 of the TA2, it became evident that some commonly used and also important terms are simply missing. These are summarized in Table 7 and have been added to the TA2023AG.

**Table 7:** General terms and terms of head and neck, costal, and pelvic anatomy lacking in the second edition of the Terminologia anatomica (FIPAT., 2019)

General Terms	Terms of head and neck anatomy	Terms of costal anatomy	Terms of pelvic anatomy
Amphiarthrosis	Alveoli dentales	Articulationes interchondrales	Fascia extraperitonealis
Bursa synovialis	Cementum	Crista costae	Fascia propria organi
Caput articulare	Gingiva	Processus costiformis; Processus costalis	Fascia retroprostatica; Septum rectovesicale
Facies articularis	Membrana atlantooccipitalis posterior		Fascia retrovaginalis; Septum rectovaginale
Fossa articularis	Musculus levator palpebrae superioris, Lamina profunda		Foramen ischiadicum majus
Intersectio tendinea	Musculus levator palpebrae superioris, Lamina superficialis		Foramen ischiadicum minus
Membrana fibrosa; Stratum fibrosum	Musculus orbitalis		Ligamentum extraperitoneale
Membrana synovialis; Stratum synoviale	Os incisivum; Premaxilla		
Musculus adductor	Periodontium		
Musculus abductor	Periodontium insertionis		
Musculus cutaneus	Periodontium protectionis		
Musculus dilatator	Spatium lateropharyngeum; Spatium pharyngeum laterale		
Musculus extensor	Spatium retropharyngeum		
Musculus flexor	Sutura incisiva		
Musculus opponens	Sutura zygomatico-maxillaris		
Musculus pronator	Sutura infraorbitalis		
Musculus rotator			
Musculus sphincter			
Musculus supinator			
Panniculus adiposus			
Recessus articularis			
Spatia interossei metacarpi			
Synarthrosis			
Vagina synovialis			

What are the intended and actual improvements of the TA2? As real improvement, the thorough use of full terms instead of short terms as usual in the TA 98 can be seen. For instance, “Paries lateralis orbitae” is better than “Paries lateralis” which is then only defined as a sub-item of the term orbita. Moreover, it can be advantageous to group osteology, arthrology and myology in one and the same part, i.e. Part II—musculoskeletal system. Third, the more or less random use of the singular and plural present in the TA 98 was largely abandoned (ten Donkelaar and Gobée 2020). But what about the proposed machine readability of the new arranged terms which should have been one of the main goals (Neumann et al. 2017)? A terminology fulfilling this prerequisite has to obey five rules as follows (Baud 2022):

1. as a major principle, each individual term must be explicit;
2. each term must have a computer-generated unique blind identifier;
3. the implemented hierarchies must be formal defined;
4. numerous anatomical textbooks use the existing—not invented—traditional Latin and—as shown above—Greek and even Arabic terms. Thus, they may not be altered beyond recognition or abandoned;
5. terminology should follow the principle of non-redundancy, i.e. a term should only be repeated once in the data base.

As apparently, the TA2 does not consequently follow these rules, readability and interpretation of the terms by machines seem at least questionable. For neither are all the terms explicit nor are they unambiguously assigned by a unique blind identifier. For example, the interosseous and lumbricales muscles have been numbered but not the cervical vertebrae. Moreover, although blind identifiers have been introduced already in 2013 for the TA98 and have also been available since that time, they have been replaced in the TA2 simply by line numbers. As a further advantage, the old identifiers were matched to the former 11-digit identifiers and thus changes in terminology could be easily tracked. Unfortunately, this is not possible anymore. Moreover, as the new identifiers, i.e. the line numbers, are already used by general data bases opened also to laypersons (as, e.g. Wikipedia), this may even lead to problematic misunderstandings (Baud 2022).

Concerning the clear definition of terminological hierarchies it has to be said that the opportunity to solve that “traditional” problem of anatomic terminology has been missed by the TA2. Instead, it presents a hierarchy of terms partly based on unspecified relations. Thus, it is not distinguished in every instance which term denominates a part of another structure or means an individual structure (Baud 2022).

The problems arising by the linguistically—and inconstantly—altered terms have already been addressed above. A pure entanglement of words will never improve any solution. Finally, non-redundancy of terms apparently remains an unresolved problem. But considering the relations of anatomical structures it is questionable if this problem can be solved. The more, machine readability is depending on strictly observing the four other rules as stated above. Thus, as seemingly the TA2 is not entirely interpretable by computers, this is—if at all—an imagined improvement.

## Resumé

Considering all these shortcomings, the FIPAT should feel addressed with regard to a new and fundamental revision of the Terminologia anatomica. In the spirit of the Accademia della Crusca proposed by Hyrtl (Hyrtl 1880, 1889) and realised earlier, anatomists from countries that primarily use Latin terminology should be involved in this process. And the compilation of the TA2023AG was not only the result of the urgent need to create a usable anatomical terminology for these countries but should also be seen as a stimulus for reflection to avoid a step back to proverbial Bable in anatomical nomenclature.

## Data Availability

Most of the data used for this article can be found in freely accessible online libraries.
